# Germination of Seeds from Flowers along a Continuum of Long to Short Styles in the Cold Desert Perennial Herb *Ixiolirion songaricum*

**DOI:** 10.3390/plants11111452

**Published:** 2022-05-30

**Authors:** Juanjuan Lu, Haiyan Yi, Dunyan Tan, Carol C. Baskin, Jerry M. Baskin

**Affiliations:** 1 College of Life Sciences, Xinjiang Agricultural University, Urümqi 830052, China; lujuanjuan@xjau.edu.cn (J.L.); carol.baskin@uky.edu (C.C.B.); jerry.baskin@yahoo.com (J.M.B.); 2 Key Laboratory of Ministry of Education of Grassland Resources and Ecology in Western Arid Region, College of Grassland Science, Xinjiang Agricultural University, Urümqi 830052, China; 3 Xinjiang Key Laboratory of Grassland Resources and Ecology, College of Grassland Science, Xinjiang Agricultural University, Urümqi 830052, China; 4 College of Biology and Geography Sciences, Kashgar University, Kashgar 844008, China; 5 Department of Biology, University of Kentucky, Lexington, KY 40506, USA; 6 Department of Plant and Soil Sciences, University of Kentucky, Lexington, KY 40506, USA

**Keywords:** bet-hedging strategy, continuous variation in style length, germination, physiological dormancy, seed size/mass

## Abstract

We compared seed set, mass, and dormancy/germination of seeds from flowers at three points on the style-length continuum [long (LS), intermediate (IS), and short (SS) styles] in *Ixiolirion songaricum*. The effects of open and hand pollination (self and cross with pollen from upper and lower-level stamens) on seed set, mass, and dormancy/germination were assessed. Most freshly-matured seeds from LS, IS, and SS flowers were dormant, and dormancy was broken under laboratory and field conditions. After-ripened seeds from LS and IS flowers germinated to significantly higher percentages than those from SS flowers. In all pollination treatments, seed set and mass were significantly higher for LS and IS than for SS flowers. Seed set, mass, and germination for LS, IS, and SS flowers were significantly higher in open-pollinated and in cross-pollinated with pollen from upper and lower-level stamens than in self-pollination with pollen from upper- and lower-level stamens. These differences in offspring reproductive traits may be adaptive for *I. songaricum* in its rainfall-unpredictable environment. This is the first study to demonstrate the association between style length and germination in a species with continuous variation in style length.

## 1. Introduction

Five classes (kinds) of stylar polymorphism are recognized by Barrett and colleagues [1,2]: heterostyly (distyly and tristyly), stylar-height polymorphism, enantiostyly, and stylar monomorphism (continuous variation in stigma height). Heterostylous populations are composed of individuals with two (distyly) or three (tristyly) floral morphs that differ in stigma and anther heights. Stylar-height polymorphism populations have individuals with two floral morphs that differ principally in the heights of stigmas. In plants with enantiostyly, the style is deflected to the left (left-styled) or right (right-styled) side of the main axis of the flower; both left- and right-styled flowers occur on the same plant, in contrast to the other four kinds of stylar polymorphism. Plant populations with stylar monomorphism, the topic of our study, consist of individuals with floral morphs that differ in the height of stigmas from low to high along a continuum.

Studies on germination have been conducted on seeds of various species of *Narcissus* with continuous stylar variation, including *N. alacaracensis* [3,4], *N. bulbocodium* [5], *N. eugeniae* [6], *N. hispanicus* [7], *N. longispathus* [4,8], *N. pseudonarcissus* [9,10,11], *N. radinganorum* [12], and *N. yepesii* [13]. However, these studies were done on a composite seed collection from flowers with different style lengths. Heretofore, no study has determined if there are differences in germination of seeds from flowers in a species with continuous variation in style length. If there is a relationship between the continuum of long to short styles and seed dormancy/germination, then the production of seeds by flowers with different style lengths that vary in degree of dormancy may serve as a bet-hedging strategy [14].

**Figure 1:**
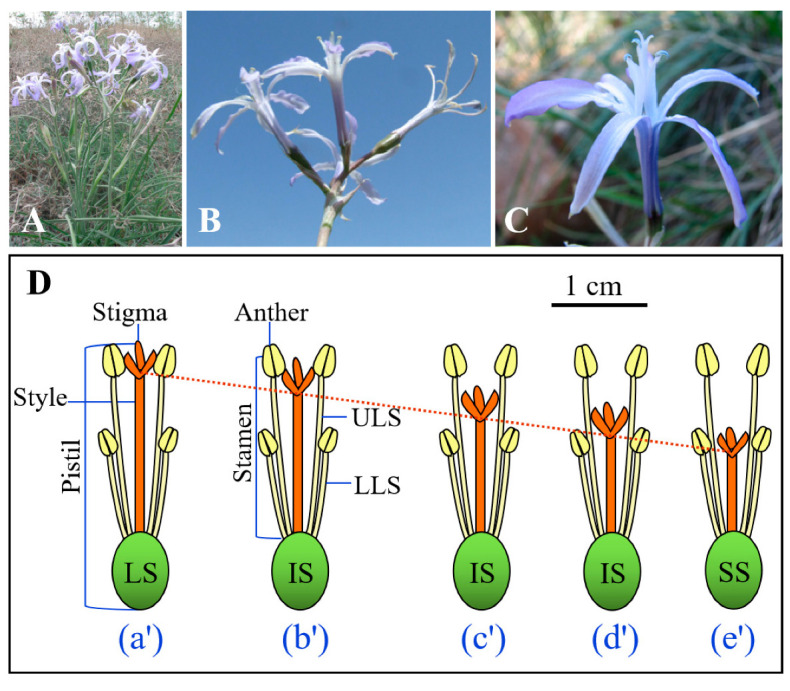
(**A**) Several plants in flower, (**B**) inflorescence of a single plant, (**C**) a flower and (**D**) diagrams illustrating continuous variation in style length for *Ixiolirion songaricum*. (**a’**) Plant with a single floral phenotype in which stigma and anthers of long stamens are of equivalent height long style (LS). Three plants (**b’**–**d’**), each with a single floral phenotype, in which stigma and anthers are spatially separated (herkogamy), intermediate style (IS). (**e’**) Plant with a single floral phenotype in which stigma and anthers of short stamens are of equivalent height short style (SS). ULS, upper-level stamen; LLS, lower-level stamen. The red-dashed line shows the style length continuum.

*Ixiolirion songaricum* P. Yan (Ixiolirionaceae) is a cold desert early-spring ephemeral-perennial herbaceous species that occurs only in the southern part of the Junggar Basin, Xinjiang Province, China [15,16]. It is mainly distributed on dry northern slopes of the Tianshan Mountain at altitudes of 400–1600 m [17]. This species is one of the common and endemic species that appear in early spring in this area. It flowers from late April to late May, and the fruits (capsules) mature from late May to early June, at which time the seeds are released from the dehiscent fruits. Flowering plants produce a single umbellate inflorescence with 2–8 flowers/fruits (Figure 1A,B), depending on the size of the plant, and fruit set in the field is very high (i.e., >80%) [18]. Each flower of *I. songaricum* has two whorls of stamens that are inserted at the base of the tepals (Figure 1C), and the length of the three outer stamens (i.e., lower-level stamen, LLS) is shorter than that of the three inner ones (i.e., upper-level stamen, ULS). The stigmas of multiple flowers within an individual are positioned between upper- and lower-level anthers. All flowers on an individual plant have the same style length, but in the population there is continuous variation in style length, with long (style length = upper stamen length, LS) (Figure 1Da’), intermediate (style length is intermediate between upper and lower stamen length, IS) (Figure 1Db’–Dd’), and short (style length = lower stamen length, SS) styles (Figure 1De’).

Given that *I. songaricum* produces flowers that exhibit continuous variation in style length among plants in the population (Figure 1D), we hypothesized that style length is associated with differences in various offspring reproductive traits in this species. To test this hypothesis, we compared seed set, seed mass, and seed dormancy-breaking and germination requirements of seeds produced by flowers at three positions along the continuum, namely long (LS) (Figure 1(Da’)), intermediate (IS) (Figure 1(Dc’)), and short (SS) (Figure 1(De’)) styles. Further, we hypothesized that there was an interaction between style length and self- vs. cross-pollination of the flowers on seed set, seed mass, and seed dormancy/germination. To test this hypothesis, various pollination treatments were given to LS, IS, and SS flowers in the field.

## 2. Results

### 2.1. Germination Ecophysiology

#### 2.1.1. Effect of Dry Storage (After-Ripening) on Germination

**Figure 2:**
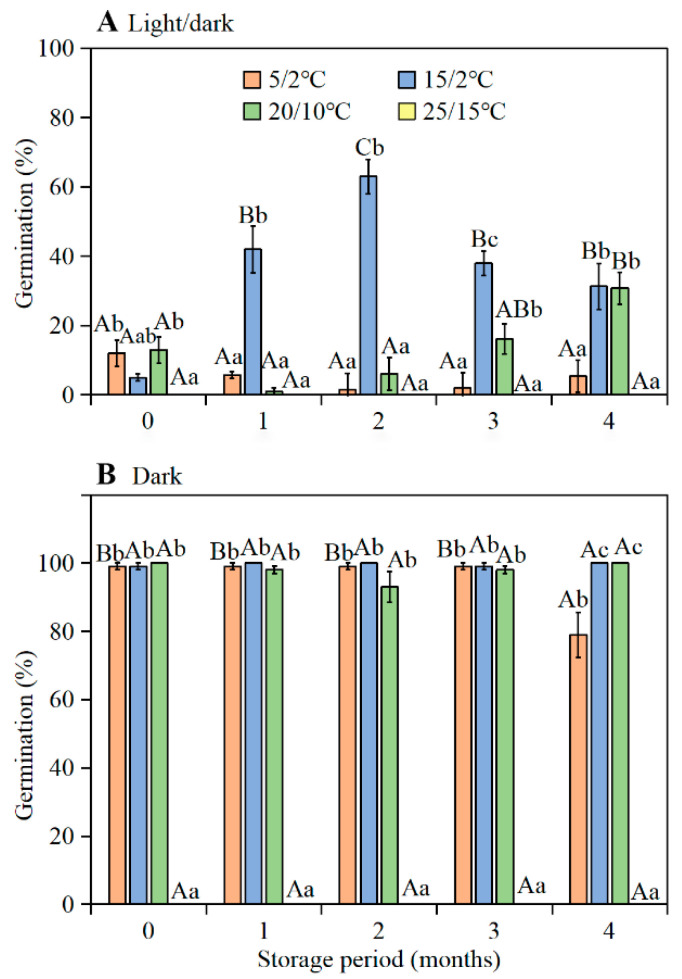
Final germination percentages (mean ± 1 s.e.) of a composite sample of seeds from flowers with long, intermediate, and short styles *of* *Ixiolirion songaricum* (**A**) in light and (**B**) in dark at four temperature regimes following 0-, 1-, 2-, 3-, and 4-months of dry storage at laboratory conditions. Bars with different uppercase letters (A, B, C) indicate significant differences among different storage times at the same temperature regimes and different lowercase letters (a, b, c) significant differences among different temperature regimes at the same storage time in the light or dark (Tukey’s HSD, *p* = 0.05).

A three-way ANOVA showed that germination was significantly affected by temperature, light, storage time, and their interactions (Supplementary Table S1). Regardless of temperature regime, only 0–13% of fresh seeds germinated in the light (Figure 2A), but 99–100% of them germinated in the dark at 5/2, 15/2, and 20/10 °C, with no germination at 25/15 °C (Figure 2B). After 1 or more months of dry storage, germination in light at 15/2 °C increased significantly to 31.3–60.0%, and after 3 months of dry storage germination in light at 20/10 °C increased significantly to 16.2–30.8%.

#### 2.1.2. Effect of Burial at Natural Temperatures on Dormancy/Germination

**Figure 3:**
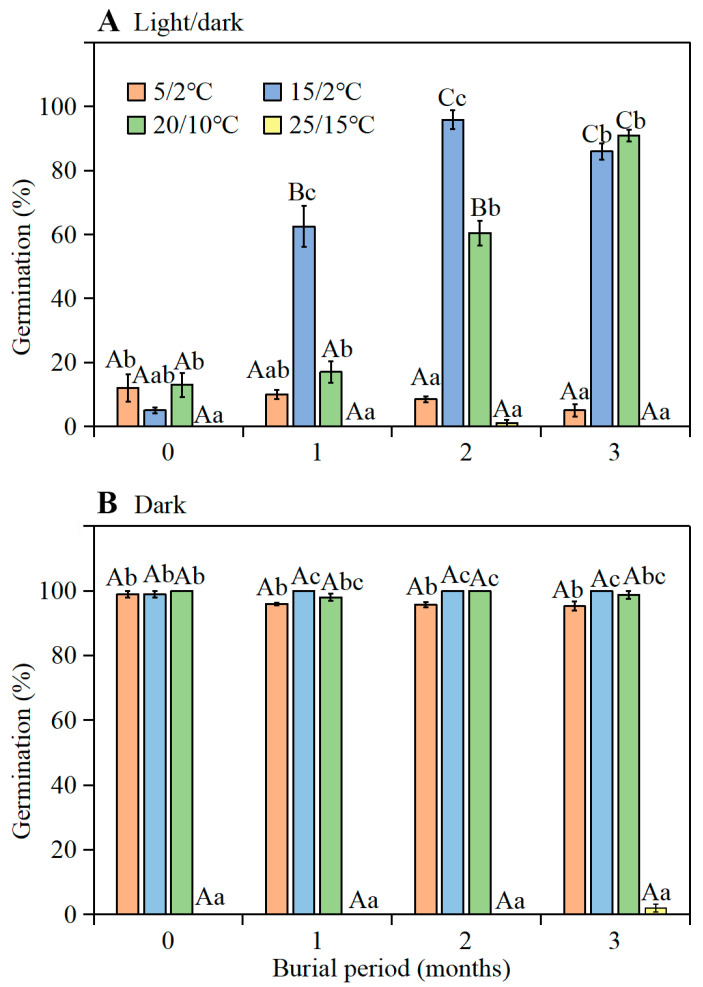
Final germination percentages (mean ± 1 s.e.) of a composite sample of seeds from flowers with long, intermediate, and short styles of *Ixiolirion songaricum* (**A**) in the light and (**B**) in the dark at four temperature regimes following 0-, 1-, 2-, and 3-months of burial in soil in experimental garden. Bars with different uppercase letters (A, B, C) indicate significant differences among different burial times at the same temperature regimes and different lowercase letters (a, b, c) significant differences among different temperature regimes at the same burial time in light or in dark (Tukey’s HSD, *p* = 0.05).

A three-way ANOVA showed that germination was significantly affected by temperature, light, retrieval time, and their interactions (Supplementary Table S2). During their burial in soil, the seeds gained the ability to germinate to high percentages in the light at 15/2 and 20/10 °C (Figure 3A). After 3 months of burial, i.e., September 2016, germination was 86% and 91% at 15/2 and 20/10 °C, respectively, in the light. In the dark, however, 99–100% of the fresh seeds germinated at 5/2, 15/2, and 20/10 °C (Figure 3B).

### 2.2. Association of Stylar Length with Seed Set, Seed Mass, and Seed Germination

#### 2.2.1. Proportion of three Stylar Lengths in the Natural Population

Percentages of plants in the natural population of *I. songaricum* with LS, IS, and SS were 19.8%, 66.0% and 14.2%, respectively.

#### 2.2.2. Effect of Dry Storage (After-Ripening) on Germination of Seeds from Plants with the Three Style Lengths

**Figure 4:**
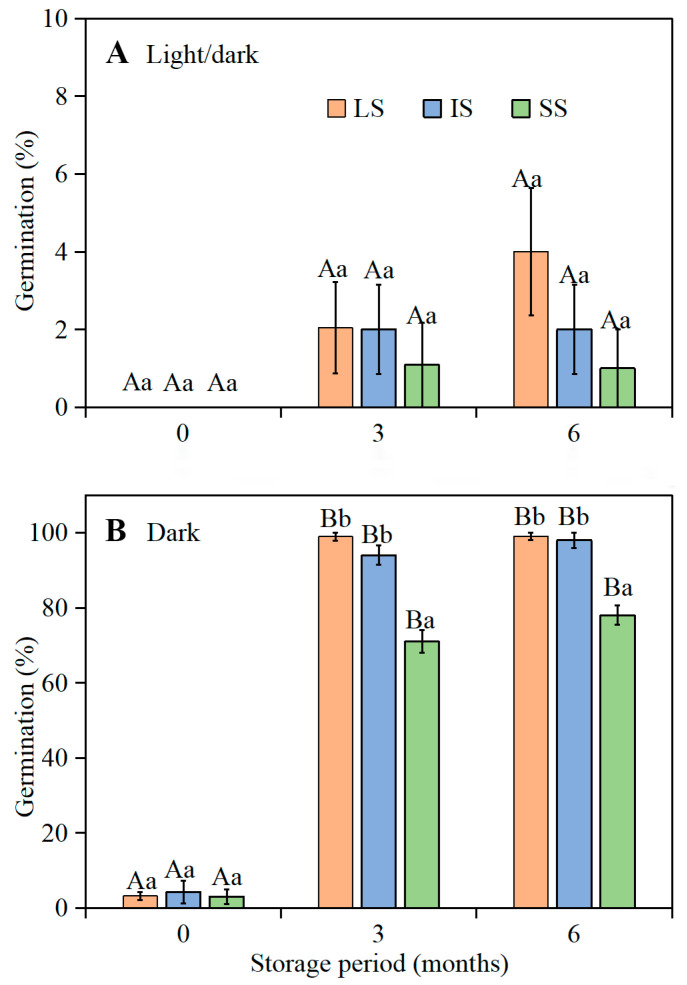
Association of style length with germination percentages (mean ± 1 s.e.) of 0-, 3-, and 6-month-old dry-stored seeds of *Ixiolirion songaricum* at 15/2 °C (**A**) in the light and (**B**) in the dark. LS, seeds from flowers with long style; IS, seeds from flowers with intermediate style; SS, seeds from flowers with short style. Bars with different uppercase letters (A, B) indicate significant differences among different style lengths at the same storage time and different lowercase letters (a, b) significant differences among different storage times at the same style length in the light or in the dark (Tukey’s HSD, *p* = 0.05).

A three-way ANCOVA showed that germination percentage was significantly affected by style length, light, storage time, and their interactions (Supplementary Table S3). Fresh seeds from flowers with LS, IS, and SS did not germinate in the light at 15/2 °C (Figure 4A), and in the dark they germinated to only 3.2%, 4.3%, and 3.0%, respectively (Figure 4B). After 3 and 6 months of dry storage, germination in light and in dark of LS, IS, and SS seeds had increased, but germination was significantly higher in dark than in light (*p* < 0.05). After 6 months of storage, LS, IS, and SS seeds incubated in darkness germinated to 99.0%, 98.0%, and 78.0%, respectively, but to only 4.0%, 2.0%, and 1.0%, respectively, in the light.

#### 2.2.3. Association of Style Length with Seed Set, Seed Mass, and Seed Germination in Pollination Experiment

**Figure 5:**
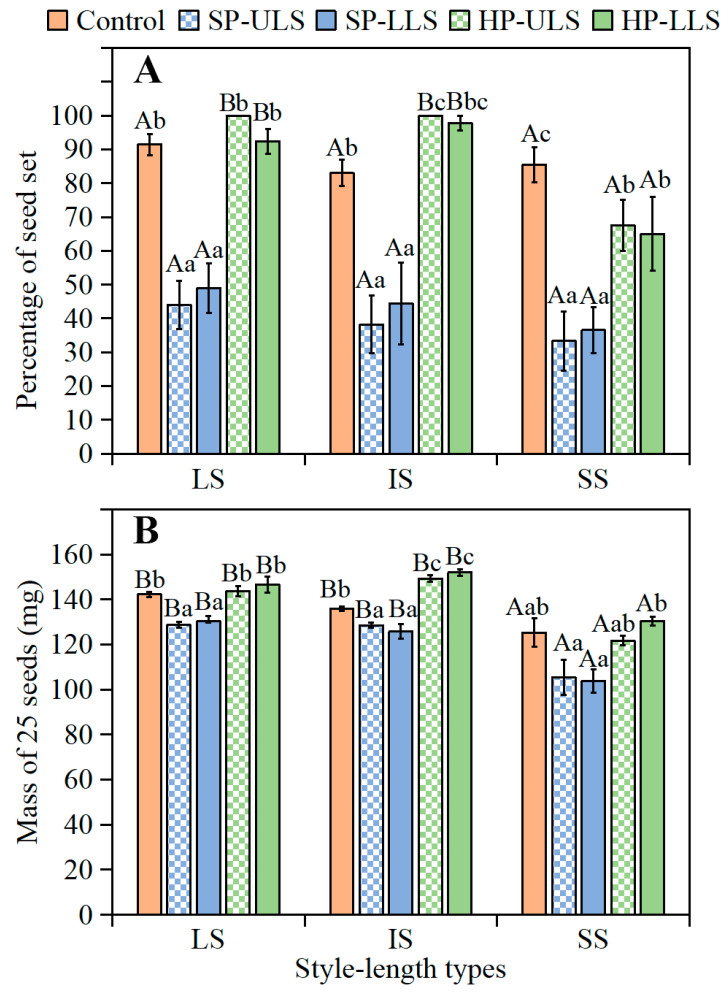
Effect of pollination treatment on flowers with different style lengths on (**A**) seed set and (**B**) seed mass of *Ixiolirion songaricum* (mean ± 1 s.e.). LS, seeds from flowers with long style; IS, seeds from flowers with intermediate style; SS, seeds from flowers with short style; Control, natural (open) pollination; SP-ULS, self-pollination with pollen from upper-level stamens; SP-LLS, self-pollination with pollen from lower-level stamens; HP-ULS, hand pollination between flowers on different plants with pollen from upper-level stamens; HP-LLS, hand pollination between flowers on different plants with pollen from lower-level stamens. Bars with different uppercase letters (A, B) indicate significant differences among different style lengths in the same treatment of artificial pollination and different lowercase letters (a, b, c) significant differences among different treatments of artificial pollination at the same style length (Tukey’s HSD, *p* = 0.05).

Seed set and seed mass. Two-way ANOVAs showed that percentages of seed set and seed mass were significantly associated with style length, pollination treatment, and their interactions (Supplementary Table S4). In all pollination treatments, flowers with LS and IS had a significantly higher percentage of seed set (Figure 5A) and seed mass (Figure 5B) than flowers with SS. For flowers with LS, IS, and SS, the percentages of seed set (Figure 5A) and seed mass (Figure 5B) were significantly higher in control and HP-ULS and HP-LLS treatments than in the SP-ULS and SP-LLS treatments. Additionally, style length was significantly negatively correlated with the percentage of seed set (r = −0.19, df = 721, *p* < 0.05) and seed mass (r = −0.53, df = 94, *p* < 0.05).

**Figure 6:**
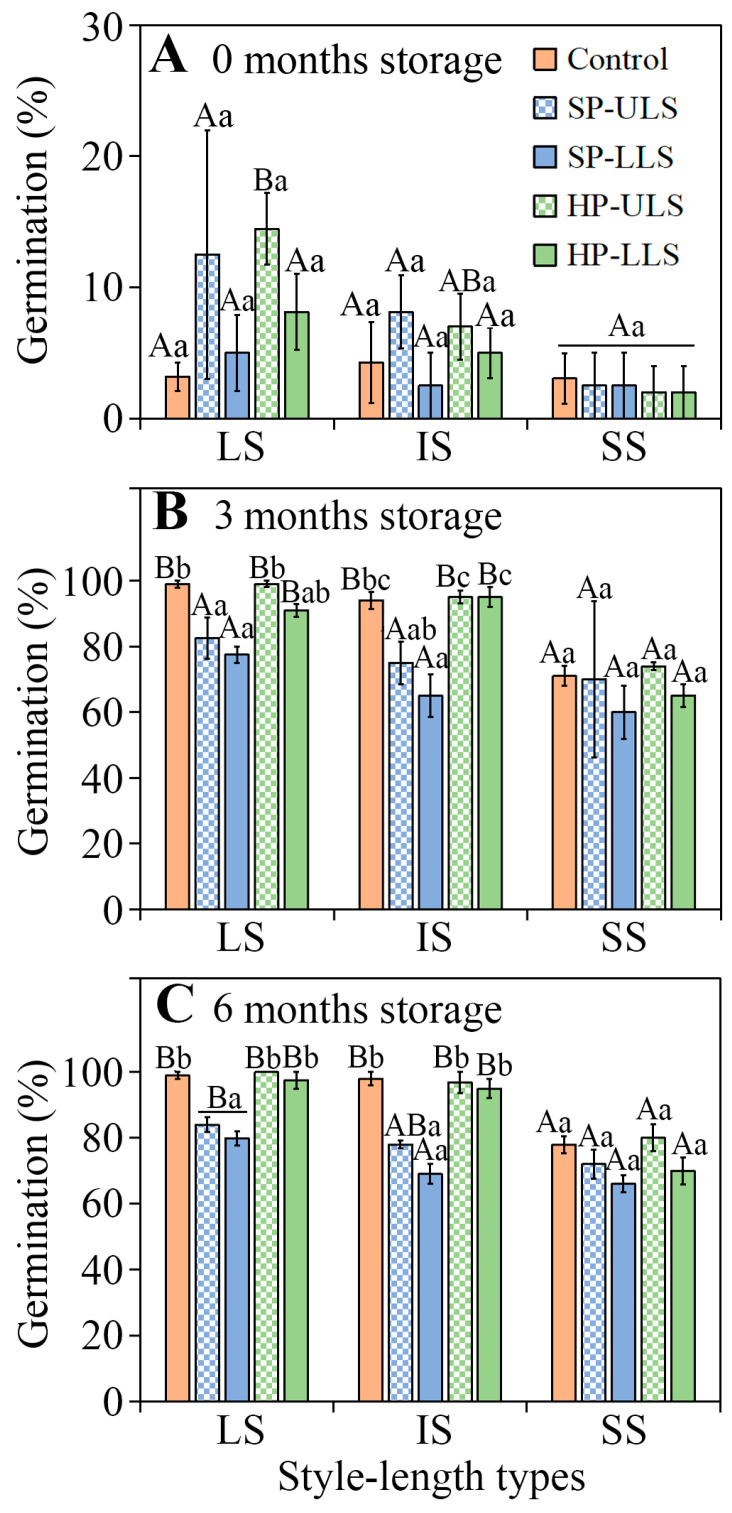
Effect of pollination treatment on final germination percentages *of Ixiolirion songaricum* seeds from flowers with different style lengths at 15/2 °C in dark following (**A**) 0-months, (**B**) 3-months, and (**C**) 6-months of dry storage at laboratory conditions (mean ± 1 s.e.). LS, seeds from flowers with long style; IS, seeds from flowers with intermediate style; SS, seeds from flowers with short style; Control, natural (open) pollination; SP-ULS, self-pollination with pollen from upper-level stamens; SP-LLS, self-pollination with pollen from lower-level stamens; HP-ULS, pollination between flowers on different plants with pollen from upper-level stamens; HP-LLS, pollination between flowers on different plants with pollen from lower-level stamens. Bars with different uppercase letters (A, B) indicate significant differences among different style lengths in the same treatment of hand pollination and different lowercase letters (a, b, c) significant differences among different treatments of hand pollination at the same style length (Tukey’s HSD, *p* = 0.05).

Seed germination. A three-way ANCOVA indicated that germination percentage was significantly associated with style length, pollination treatment, storage time, the interaction between style length, and storage time and interaction between pollination treatment and storage time (Supplementary Table S5). Regardless of pollination treatment, the highest germination of fresh seeds from flowers with LS, IS, and SS was only 14.5, 8.1, and 3.0%, respectively (Figure 6A). Seeds from all pollination treatments of flowers with LS, IS, and SS after-ripened rapidly during storage (Figure 6B). In control and HP-ULS and HP-LLS treatments, germination percentages of 3- (Figure 6B) and 6-month-stored (Figure 6C) seeds from plants with LS and IS were significantly higher than that of seeds from plants with SS. However, germination did not differ significantly among seeds from plants with LS and IS, and in the SP-ULS and SP-LLS treatments germination did not differ significantly among seeds from plants with LS, IS, and SS.

## 3. Discussion

Our hypothesis that style length is associated with offspring reproductive traits of *I. songaricum* was confirmed via a comparison of seed set, seed mass, and seed dormancy-breaking/germination requirements for seeds from plants along the continuum of style lengths. The presence of flowers with continuous variation in style length among plants in the population not only promoted out-crossing success [18], but it was also associated with reproductive traits (including seed mass and size) and germination in our study. Ours is the first study to document the association between seed germination and style length in a species with continuous variation in style length.

Freshly-matured seeds (mixed sample) of *I. songaricum* germinated to high percentages in the dark at 5/2, 15/2, and 20/10 °C, but to low percentages in the light at these temperature regimes. Following 1–2 months of after-ripening under laboratory conditions and burial in the experimental garden during summer, germination of seeds incubated in the light increased greatly. Since (1) the embryo of *I. songaricum* is fully developed, and (2) dormancy was broken during dry storage, we conclude that seeds have non-deep physiological dormancy. Further, since the range of conditions over which seeds germinated to a high percentage increased as seeds after-ripened, they were in conditional (non-deep) physiological dormancy when they first matured [19,20].

The highest germination of seeds buried in the experimental garden was 91.0% (in the light at 20/10 °C) and 100.0% (in the dark at 15/2 °C) when exhumed and tested in September. Thus, optimum temperatures for germination of seeds buried in the garden in autumn were 15/2 and 20/10 °C. These results are consistent with the germination responses reported for seeds of *I. songaricum* [21] and *I. tataricum* [22], in which the germination percentage was higher at low than at high temperatures. In addition, similar to the seeds of *I. tataricum* [22], those of *I. songaricum* germinate to higher percentages in darkness than in the light. Although seeds *of I. songaricum* in the experimental garden can germinate to high percentages when tested at low temperatures in autumn, in most years they do not germinate in the field/garden in autumn due to the low amount of rainfall. Seedlings of *I. songaricum* have been observed in the garden and in the field only in spring when soil moisture is high due to rainfall and snowmelt (JL, unpublished). Germination of *I. tataricum* seeds did not occur in high water stress [23].

Unlike the seeds of temperate zone obligate winter annuals that germinate only in autumn, those of *I. songaricum* are not induced back into dormancy (secondary dormancy) by low temperatures during winter. Most freshly-matured seeds from LS, IS, and SS flowers of *I. songaricum* produced in 2019 were dormant, and thus germination was zero or very low at the optimum temperature regime in both light and darkness. In contrast, the composite of fresh LS, IS, and SS seeds from 2016 had conditional physiological dormancy and germinated to high percentages in the dark but not in the light. These results suggest that there is year-to-year variation in depth of dormancy of fresh seeds of *I. songaricum*. After 3 to 6 months of after-ripening under laboratory conditions, the 2019 *I. songaricum* seeds from LS, IS, and SS flowers germinated to high percentages in darkness but not in light. However, in darkness, the 3- and 6-month-old seeds from flowers with SS germinated to significantly lower percentages than those from flowers of LS and IS. In the heterostylous (tristylous) emergent aquatic species *Pontederia cordata*, germination of seeds from flowers with short-, mid-, and long-styles did not differ significantly [24].

After 3 and 6 months of after-ripening in dry storage, the SS seeds of *I. songaricum* from natural (open) pollinated flowers and those from the HP-US and HP-LS pollination treatments had significantly lower germination percentages than seeds from plants with LS and IS. In the SP-US and SP-LS treatments, however, germination did not differ significantly among the seeds from flowers with LS, IS, and SS in this species. For the heterostylous (tristylous) wetland emergent weed *Lythrum salicaria*, seeds of legitimate hand-pollinated full-sib families from SS flowers germinated to significantly higher percentages than those from IS and LS flowers, which did not differ from each other [25]. Also, seeds of legitimate hand-pollinated full-sib families from SS flowers of *L. salicaria* germinated to significantly higher percentages than those from IS and LS flowers, which did not differ from each other, except seeds from IS flowers germinated to a higher percentage than those from LS flowers [25].

In the natural population, percentage of *I. songaricum* plants with the three style lengths was IS > LS > SS. Moreover, there were significant differences for seed mass [(LS > IS) > SS] in all pollination treatments and for the percentage of seed set in HP-ULS and HP-LLS treatments among the plants with the three style lengths. Seed mass is an important biological trait affecting seed germination [26]. Small seeds from plants with SS styles of *I. songaricum* had a lower germination percentage than the larger seeds from plants with LS and IS styles, suggesting a positive effect of seed mass on germination, as has been found for other species [27,28,29,30]. Further, with seed mass as a covariate germination percentage also differed significantly among 3- and 6-month dry storage of *I. songaricum* seeds from plants with LS and IS styles. Thus, the germination of seeds from plants with LS and IS styles was different, although the seeds did not differ significantly in initial size.

On the other hand, the effects of seed mass from flowers with continuous variation in style length are very important for the germination of this species and may be of adaptive significance in the establishment and growth of seedlings and maintaining populations. It has been documented in other species that seedlings from large seeds are able to survive hazards better than those from small seeds [29,31,32], including stressful environmental conditions such as drought, soil/sand burial, and low nutrient availability. However, the small seeds produced by flowers with the SS style may be a part of the adaptive life history strategy of *I. songaricum* to its cold desert habitat. Thus, the combination of small seeds produced by SS flowers that are more dormant and the large seeds produced by LS and IS flowers that are less dormant might serve the same role in maintaining the species in its habitat as the production of two or more kinds of seeds in heterodiasporous [33] and amphicarpic [34] species.

Production of *I. songaricum* seeds from plants with continuous style lengths that differ in mass and depth of dormancy within a population may enable this species to bet-hedge in the unpredictable rainfall environment of the Junggar Desert. On one end of the continuum, delay of germination of small seeds from SS flowers constitutes a low-risk means of reproduction, and thereby should increase the probability of population persistence of this species. On the other end of the continuum, large seeds from LS flowers constitute a high-risk strategy that should be adaptive in a good year for germination and plant establishment.

In summary, the seeds from plants with continuous variation in style length of the cold desert perennial species *I. songaricum* exhibit continuous variation in seed mass and germination ecology. Thus, the small seeds (from SS flowers) have the highest degree of dormancy, while the large seeds (from LS flowers) have the lowest degree of dormancy. Theoretically, this “cryptic continuous variation in style lengths” is likely an adaptation to the unpredictable environment of the mother plant. However, ecological studies are needed to confirm (or not) that this theory applies to the species *I. songaricum* with continuous variation in style length in its cold desert environment.

## 4. Materials and methods

### 4.1. Field Site Description

The field study site is a gravelly desert on Yamalic Hill in a suburb of Urümqi city near the southern edge of the Junggar Basin of Xinjiang Uyghur Autonomous region, China (43°22′−44°25′ N, 80°58′−87°47′ E, 900–1100 m a.s.l.). For the years 1991–2019 at Urümqi, the mean annual temperature was 7.7 °C, and the mean monthly temperatures of the coldest (January) and hottest (July) months were −15.7 °C and 28.9 °C, respectively. The average annual precipitation was 264.2 mm and ranged from 48.3 to 419.5 mm; the coefficient of variation (CV = standard deviation/mean) was 38.7% (National Meteorological Information Center, China Meteorological Administration). The annual potential evaporation is >2000 mm [35].

### 4.2. Germination Ecophysiology

#### 4.2.1. Fruit Collection

Freshly-matured capsules were collected from the dry infructescences of *I. songaricum* plants in the Yamalic Hill population on 11 June 2016. In the laboratory, seeds were separated from fruits and other plant material and stored dry in paper bags at room conditions until used. This was a mixed (composite) seed collection, i.e., from flowers of different style lengths, which was used to gain preliminary information about seed dormancy and germination of the species. In addition, 10 seeds each from plants with the three style lengths were imbibed, cut open, and the embryo examined; they were fully developed. Embryo length to seed length (E:S) ratios were 0.81 ± 0.02, 0.80 ± 0.01, and 0.77 ± 0.02 for seeds from LS, IS, and SS flowers, respectively, and they did not differ significantly.

#### 4.2.2. Effect of Dry Storage (After-Ripening) on Germination

The seeds (mixed collection) were stored dry in laboratory conditions (ca. 28 °C, 46–50% RH) for 0 (fresh), 1, 2, 3, and 4 months and then tested for germination. The tests of fresh seeds (0 months old) were initiated on 13 June 2016, using seeds collected on 11 June 2016. For each test condition, four replicates of 25 seeds were incubated on two layers of Whatman No.1 filter paper moistened with 2.5 mL of distilled water in 9-cm-diameter Petri dishes. Seeds were incubated at daily (12 h light/12 h dark; hereafter light) temperature regimes of 5/2, 15/2, 20/10, and 25/15 °C in light (12 h ≈ 100 μmol·m ^−2^ s^−1^, 400–700 nm, cool white fluorescent light each day and 12 h dark) or in constant darkness (Petri dishes with seeds in them placed in light-proof black bags) for 28 d. Germination (radicle emergence) in light was examined daily for 28 d; germinated seeds were removed at each counting. Seeds incubated in darkness were checked only after 28 d. Thus, they were not exposed to any light during the incubation period. After germination trials were complete, the non-germinated seeds were tested for viability. The seeds were cut open and the embryos observed. Seeds with white and firm embryos were counted as viable, and those with soft, tan embryos were considered non-viable and excluded from the calculations of germination percentages; ≤2% of the seeds were nonviable.

#### 4.2.3. Effect of Burial at Natural Temperatures on Dormancy/Germination

Our purpose was to determine when seeds (mixed collection) germinate under field (experimental garden) conditions. Three days after collection on 11 June 2016, 1300 seeds were placed in each of 12 fine-mesh nylon bags (8 cm long and 5 cm wide). Each bag of seeds was buried at a depth of 3 cm in plastic pots (13 cm deep and 15 cm diameter with drainage holes at the bottom) filled with soil from the habitat of *I. songaricum*. The pots were placed on the soil surface in the experimental garden on the campus of Xinjiang Agricultural University in Urümqi, near the southern edge of the Junggar Basin. Seeds were subjected to natural temperatures and precipitation. Temperature data for the duration of this experiment were obtained from the National Meteorological Information Center, China Meteorological Administration.

The germination of fresh seeds (0 months old) was tested on 13 June 2016, using seeds collected on 11 June 2016. Starting on 13 July 2016 (seeds buried for 1 month), at monthly-intervals, one haphazardly selected pot of seeds was taken to the laboratory and germination tests were performed. However, on 13 October 2016 (after 4 months of burial), about 98% of the seeds in the bag had germinated, thus germination tests could not be performed. For germination tests, four dishes of 25 seeds each were incubated in 9-cm-diameter Petri dishes on filter paper in light and in darkness at 5/2, 15/2, 20/10, and 25/15 °C for 28 d, as described above.

### 4.3. Association of Stylar Length with Seed Set, Seed Mass, and Seed Germination

During the peak flowering period in late April 2019, 1000 plants were selected from the several thousand flowering individuals in the Yamalic Hill population of *I. songaricum*. Plants, each with LS, IS, or SS, were marked with a different color of knitting wool. Then, the studies were conducted as follows.

#### 4.3.1. Proportions of Three Stylar Lengths in the Natural Population

The number of plants in the three style-length categories (i.e., flowers with LS, IS, or SS styles) was recorded, and the percentage of plants in each group was calculated.

#### 4.3.2. Effect of Dry Storage (After-Ripening) on Germination of Seeds from Plants with Different Style Lengths

In early June 2019, mature fruits were collected from the marked plants, and seeds from each of 1000 plants with LS, IS, or SS were stored separately in paper bags at room conditions until used.

Seeds from the plants with the three style lengths stored dry in the laboratory for 0 (3 June 2019), 3 (3 September 2019), and 6 (3 December 2019) months were incubated in Petri dishes on wet filter paper at the optimum temperature regime (i.e., 15/2 °C) in light and dark conditions for 28 d, as described above. Four replicates of 25 seeds for each of the three style lengths were used to test germination (25 seeds × 4 replications × 3 style lengths × 3 storage times × 1 temperature regime × 2 light conditions = 1800 seeds).

#### 4.3.3. Association of Style Length with Seed Set, Seed Mass, and Seed Germination in Pollination Experiment

The purpose of this experiment was to determine if seed set, mass, and germination differed between plants with LS, IS, and SS after hand pollination. To ensure that biomass allocation to the reproductive organs of plants was not the limiting factor for any treatment, only two flowers were retained on each marked plant; the other flowers were removed. The two flowers remaining on a plant received the same pollination treatment as follows.

(1) Natural pollination. The two flowers on a plant were open-pollinated and thus served as controls.

(2) Manual pollination experiments. The two flowers on a plant were bagged before anthesis to exclude floral visitors. When the stigmatic lobes had opened completely, the bags were removed (after the natural closure of the flower), and manual pollination treatments were performed: (1) Self-pollination with pollen from upper-level stamens (SP-ULS); (2) Self-pollination with pollen from lower-level stamens (SP-LLS); (3) Pollination between flowers on different plants with pollen from the upper-level stamens (HP-ULS); and (4) Pollination between flowers on different plants with pollen from lower-level stamens (HP-LLS). For pollination between flowers on different plants, the flowers were emasculated and cross-pollinated with pollen of the upper (HP-ULS) and lower-level (HP-LLS) stamens from flowers on other plants at least 10 m away; in these crosses, the style and anthers were the same height. Once hand pollination was finished, the flowers were rebagged until they produced mature fruits.

A total of 3000 flowers were used in this experiment [100 plants × 2 flowers per plant × 5 treatments (i.e., 1 natural pollination + 4 manual pollinations) × 3 style lengths = 3000 flowers]. Mature fruits were collected from all treatments on 28 May 2019, before seed dispersal. Fruits from each treatment were stored in a separate paper bag at room conditions (ca. 28 °C, 46–50% RH) until used.

Seed set and seed mass. When fruits were harvested, the number of flowers with LS, IS, and SS in each pollination treatment that produced seeds was recorded and the percentages of seed set calculated. Ten replications of 25 seeds each from plants with LS, IS, and SS in each treatment (10 replications × 25 seeds × 3 style lengths × 5 treatments) stored dry under laboratory conditions for 7 days were weighed using a Sartorius BS210S electronic balance (0.0001 g).

Seed germination. Seeds from LS, IS, and SS plants in each treatment were stored dry in the laboratory for 0 (29 May 2019), 3, and 6 months and incubated in Petri dishes on wet filter paper at the optimum condition for germination (15/2 °C in dark) for 28 d, as described above. Four replicates of 25 seeds from LS, IS, and SS plants in each treatment were used to test germination (25 seeds × 4 replications × 3 style lengths × 5 treatments × 3 storage time = 4500 seeds).

### 4.4. Statistical Analysis

All data were analyzed for normality and homogeneity of variance prior to analysis to fulfill the requirements of one-way analysis of variance (ANOVA). Percentage data were arcsine transformed before statistical analysis to ensure homogeneity of variance (non-transformed data appear in all figures). If data were not normally distributed or if variances were not homogeneous, they were arcsine (square root %) (percentage data) or log_10_ (other data) transformed before analysis to ensure homogeneity of variance. When the variance of logarithmically transformed data was still not homogenous, differences among treatments were determined by the non-parametric Kruskal–Wallis test. Tukey’s HSD test was performed for multiple comparisons to determine significant (*p* < 0.05) differences among the treatments.

When variances of non-transformed or transformed data were homogeneous, a one-way ANOVA was used to determine differences in germination percentages of seeds stored in the laboratory and buried in soil among temperature regimes and among periods of time. Also, ANOVA was used to determine differences in percentages of fruit and seed set, number of seeds per fruit, seed mass, and germination percentage among seeds from plants of the three groups (LS, IS, and SS) and among the treatments of artificial pollination (i.e., control, HP-ULS, HP-LLS, SP-ULS, and SP-LLS).

Three-way ANOVAs were used to test for significance of main effects (temperature, light, and storage time) and their interactions on germination in the “Effect of dry storage (after-ripening) on germination” experiment and for significance of main effects (temperature, light, and retrieval time) and their interactions on germination in the “Effect of burial at natural temperatures on dormancy/germination” experiment. Two-way ANOVAs were used to test for significance of main effects (temperature and style length) and their interactions on seed set and seed mass in the “Association of style length with seed set and seed mass in pollination experiment”.

With seed mass as a covariate, three-way ANCOVAs were used to test for significance of main effects (type of variation in style length, light, and storage time) and their interactions on germination in the “Effect of dry storage (after-ripening) on germination of seeds from plants with different style lengths” experiment and for significance of main effects (type of variation in style length, treatment of artificial pollination, and storage time) and their interactions on germination in the “Association of style length with seed germination in artificial pollination experiment”. Statistical tests were conducted at *p* < 0.05.

Correlative analyses were used to determine the relationships between style length (LS, IS, and SS) and seed set, and seed mass, and between artificial pollination treatments and seed set and seed mass. All data analyses were performed with the software SPSS 19.0 (SPSS Inc., Chicago, IL, USA). Values are means ± 1 s.e.

## Data Availability

Not applicable.

## Supplementary Materials

The following supporting information can be downloaded at: https://www.mdpi.com/article/10.3390/plants11111452/s1, Table S1: A three-way ANOVA of effects of temperature (T), light (L), storage time (S), and their interactions on germination of *Ixiolirion songaricum* of seeds stored dry at laboratory conditions. Table S2: A three-way ANOVA of effects of temperature (T), light (L), retrieval time (R), and their interactions on germination of *Ixiolirion songaricum* of seeds stored (buried) in soil in the experimental garden. Table S3: A three-way ANCOVA of effects of style length (T), light (L), storage time (S), and their interactions on germination of *Ixiolirion songaricum* of seeds stored dry at laboratory conditions, with seed mass (SM) as a covariate. Table S4: A two-way ANOVA of effects of variation in style length (T), treatment (T’), and their interaction on percentage of fruit and seed set, number of seeds per fruit, and seed mass of *Ixiolirion songaricum*. Table S5: A three-way ANCOVA of effects of style length (T), treatment (T’), storage time (S), and their interactions on germination of *Ixiolirion songaricum* seeds stored dry at laboratory conditions, with seed mass (SM) as a covariate.
