# Supplementary material for: Germination of Seeds from Flowers along a Continuum of Long to Short Styles in the Cold Desert Perennial Herb Ixiolirion songaricum
Source: Plants (Basel). 2022 May 30;11(11):1452. doi: 10.3390/plants11111452 (PMC9182588; doi:10.3390/plants11111452)
Supplement: Supplementary file 1 [file plants-11-01452-s001.zip › plants-1691582-supplementary.pdf]

plants

Table S1: A three-way ANOVA of effects of temperature (T), light (L), storage time (S), and their interactions on germination of *Ixiolirion songaricum* of seeds stored dry at laboratory conditions.

Source	d.f.	SS	MS	F-value	P-value
Temperature (T)	3	106813.43	35604.48	1143.00	< 0.05
Light (L)	1	141365.49	141365.49	4539.00	< 0.05
Storage time (S)	4	315.40	78.85	2.53	< 0.05
T × L	3	50878.66	16959.55	544.49	< 0.05
T × S	12	5464.13	455.34	14.62	< 0.05
L × S	4	994.36	248.59	7.98	< 0.05
T × L × S	12	4005.52	333.79	10.72	< 0.05

Table S2: A three-way ANOVA of effects of temperature (T), light (L), retrieval time (R), and their interactions on germination of *Ixiolirion songaricum* of seeds stored (buried) in soil in the experimental garden.

Source	d.f.	SS	MS	F-value	P-value
Temperature (T)	3	125432.23	41810.74	2439.00	< 0.05
Light (L)	1	64158.10	64158.10	3743.00	< 0.05
Retrieval time (R)	3	7338.97	2446.32	142.72	< 0.05
T × L	3	31638.39	10546.13	615.28	< 0.05
T × R	9	11284.18	1253.80	73.15	< 0.05
L × R	3	7601.49	2533.83	147.83	< 0.05
T × L × R	9	10469.98	1163.33	67.87	< 0.05

Table S3: A three-way ANCOVA of effects of style length (T), light (L), storage time (S), and their interactions on germination of *Ixiolirion songaricum* of seeds stored dry at laboratory conditions, with seed mass (SM) as a covariate.

Source	d.f.	SS	MS	F-value	P-value
Seed mass (SM)	1	2.73	2.73	0.24	0.63
Type of variation in style length (T)	2	1112.01	556.01	47.76	< 0.05
Light (L)	1	64159.05	64159.05	5511.43	< 0.05
Storage time (S)	2	31278.58	15639.29	1343.46	< 0.05
T × L	2	852.59	426.30	36.62	< 0.05
T × S	4	531.69	132.92	11.42	< 0.05
L × S	2	28458.90	14229.45	1222.35	< 0.05
T × L × S	4	433.98	108.50	9.32	< 0.05

Table S4: A two-way ANOVA of effects of variation in style length (T), treatment (T'), and their interaction on percentage of fruit and seed set, number of seeds per fruit, and seed mass of *Ixiolirion songaricum*.

Source	d.f.	SS	MS	F-value	P-value
Percentage of seed set					
T	2	45237.09	22618.55	16.24	< 0.05
T'	4	271428.58	67857.15	48.72	< 0.05
T × T'	8	24984.85	3123.11	2.24	< 0.05
Seed mass					
T	2	6568.02	3284.01	40.53	< 0.05
T'	4	8334.49	2083.62	25.72	< 0.05
T × T'	8	2085.84	260.73	3.22	< 0.05

Table S5: A three-way ANCOVA of effects of style length (T), treatment (T'), storage time (S), and their interactions on germination of *Ixiolirion songaricum* seeds stored dry at laboratory conditions, with seed mass (SM) as a covariate.

Source	d.f.	SS	MS	F-value	P-value
Seed mass (SM)	1	98.86	98.86	1.01	0.32
Type of variation in style length (T)	2	3740.86	1870.43	19.18	< 0.05
Treatment of artificial pollination (T')	4	3244.25	811.06	8.32	< 0.05
Storage time (S)	2	237859.23	118929.61	1219.46	< 0.05
T × T'	8	1001.47	125.18	1.28	0.26
T × S	4	1640.69	410.17	4.21	< 0.05
T' × S	8	2513.62	314.20	3.22	< 0.05
T × T' × S	16	858.53	53.66	0.55	0.91
